# Virulence Diversity among Bacteremic *Aeromonas* Isolates: *Ex Vivo*, Animal, and Clinical Evidences

**DOI:** 10.1371/journal.pone.0111213

**Published:** 2014-11-06

**Authors:** Po-Lin Chen, Chi-Jung Wu, Pei-Jane Tsai, Hung-Jen Tang, Yin-Ching Chuang, Nan-Yao Lee, Ching-Chi Lee, Chia-Wen Li, Ming-Chi Li, Chi-Chung Chen, Hung-Wen Tsai, Chun-Chun Ou, Chang-Shi Chen, Wen-Chien Ko

**Affiliations:** 1 Department of Internal Medicine, National Cheng Kung University Hospital, Tainan, Taiwan; 2 Department of Pathology, National Cheng Kung University Hospital, Tainan, Taiwan; 3 Graduate Institute of Clinical Medicine, National Cheng Kung University College of Medicine, Tainan, Taiwan; 4 Department of Medical Laboratory Science and Biotechnology, National Cheng Kung University College of Medicine, Tainan, Taiwan; 5 Department of Biochemistry and Molecular Biology, National Cheng Kung University College of Medicine, Tainan, Taiwan; 6 Department of Medicine, National Cheng Kung University College of Medicine, Tainan, Taiwan; 7 National Institute of Infectious Diseases and Vaccinology, National Health Research Institutes, Taipei, Taiwan; 8 Research Center of Infectious Disease and Signaling, National Cheng Kung University, Tainan, Taiwan; 9 Department of Medicine, Chi Mei Medical Center, Tainan, Taiwan; 10 Department of Medical Research, Chi Mei Medical Center, Tainan, Taiwan; 11 Department of Clinical Pathology, Chi Mei Medical Center, Tainan, Taiwan; 12 Department of Health and Nutrition, Chia Nan University of Pharmacy and Science, Tainan, Taiwan; Leibniz Institute for Natural Products Research and Infection Biology- Hans Knoell Institute, Germany

## Abstract

**Background:**

The objective of this study was to compare virulence among different *Aeromonas* species causing bloodstream infections.

**Methodology/Principal Findings:**

Nine of four species of *Aeromonas* blood isolates, including *A. dhakensis*, *A. hydrophila*, *A. veronii* and *A. caviae* were randomly selected for analysis. The species was identified by the DNA sequence matching of *rpoD*. Clinically, the patients with *A. dhakensis* bacteremia had a higher sepsis-related mortality rate than those with other species (37.5% *vs.* 0%, *P* = 0.028). Virulence of different *Aeromonas* species were tested in *C. elegans*, mouse fibroblast C2C12 cell line and BALB/c mice models. *C. elegans* fed with *A. dhakensis* and *A. caviae* had the lowest and highest survival rates compared with other species, respectively (all *P* values <0.0001). *A. dhakensis* isolates also exhibited more cytotoxicity in C2C12 cell line (all *P* values <0.0001). Fourteen-day survival rate of mice intramuscularly inoculated with *A. dhakensis* was lower than that of other species (all *P* values <0.0001). Hemolytic activity and several virulence factor genes were rarely detected in the *A. caviae* isolates.

**Conclusions/Significance:**

Clinical data, *ex vivo* experiments, and animal studies suggest there is virulence variation among clinically important *Aeromonas* species.

## Introduction

Aeromonads, belonging to the genus *Aeromonas*, are gram-negative rods, which can proliferate in aquatic environments and soils. They are important endemic pathogens in southern Taiwan as well as other areas worldwide [1], [2], and have been implicated in a variety of human infectious diseases, including gastroenteritis, wound infections, septicemia, respiratory infections, hepatobiliary infections, and urinary tract infections [3]. Most human diseases were reported to be associated with three species *A. hydrophila*, *A. veronii*, and *A. caviae* [4]–[7]. The reported mortality rate among patients with *Aeromonas* bacteremia varies from 24% to 63% [5]. Of note, higher case fatality rates were noted in patients with *A. hydrophila* and *A. veronii* bacteremia in the literature, ranging from 33% to 56% [5], [8], [9]. Nevertheless, clinical infections due to *A. dhakensis* were rarely described in the literature for several reasons. First, *A. dhakensis*, previously named *A. aquariorum* or *A. hydrophila* sub. *dhakensis*, was often recognized as *A. hydrophila* by the current phenotype-based identification system. Second, correct identification of *A. dhakensis* needs specific molecular methods, such as *rpoD* or *gyrB* sequencing [10]–[12]. Therefore, the importance attributed to *A. dhakensis* in human infections should be re-evaluated due to the changing taxonomy.

Morinaga *et al.* had reported that *A. dhakensis* could carry an array of virulence factors and exhibit the most potent toxicity to human blood cell lines among the tested *Aeromonas* species [11]. Our previous study also demonstrated that *A. dhakensis* isolates are more toxic to human normal skin cell lines than *A. hydrophila* isolates [12]. However, comparative studies of clinical presentations among *Aeromonas* species, including *A. dhakensis*, are not reported. Thus, our aim was to analyze the clinical presentations of bloodstream infections due to common *Aeromonas* species as well as their virulence in animal models of *Caenorhabditis elegans* and mice.

## Materials and Methods

### Bacterial isolates

The study isolates were selected from stored *Aeromonas* blood isolates between January of 2004 and April of 2011 at National Cheng Kung University Hospital, a medical center in southern Taiwan. The phenotype of species was determined by the Vitek 2 GN (bioMérieux, Inc., Durham, NC, USA) and/or API 20E (BioMérieux Marcy-l'Etoile, France) identification cards and biochemical tests. Species identification of each *Aeromonas* isolate was determined based on the partial sequences of *rpoD* as described before [13]. The GenBank accession numbers of the *rpoD* sequences for *Aeromonas* isolates are listed in the Table S1 in File S1. All *Aeromonas* isolates were stored at −70°C until use.

Nine isolates of each common *Aeromonas* species, including *A. dhakensis*, *A. hydrophila*, *A. veronii*, and *A. caviae*, were randomly selected. The reference strains for *rpoD* sequencing (GenBank accession no.) included *A. hydrophila* subsp. *dhakensis* CECT^T^ 5744 (EF465510.1), *A. hydrophila* ATCC 7966^T^ (AY127856.1), *A. veronii* CECT 4246^T^ (AY987685.1), and *A. caviae* CECT 838^T^ (AY169337). Clinical details of these 36 patients were obtained from medical charts. The study was ethically approved by The Institutional Review Board of National Cheng Kung University Hospital (IRB no. B-ER-101-031) and the requirement for informed consent was waived.

### Definitions

The medical records of the selected patients were reviewed retrospectively. The sites of infection were determined on the basis of clinical findings or bacterial culture results [9]. Acute cholangitis was diagnosed by the presence of clinical signs of right upper quadrant pain, fever, and jaundice, in addition to *Aeromonas* growth in the bile, which was collected by percutaneous transhepatic cholangiodrainage [14]. Catheter-related bloodstream infection was defined as a positive semi-quantitative tip culture (≥15 colony-forming units), bacteremia, and/or high clinical suspicion [14]. Diagnosis of spontaneous bacterial peritonitis was based on the presence of a polymorphonuclear leukocyte count of ≥250/mm^3^ in ascetic fluid, which was collected by diagnostic paracentesis, and the exclusion of secondary peritonitis [15]. Those without apparent infection sites were defined as the cases of primary bacteremia.

Sepsis-related mortality was the death of a patient with a clinical course suggestive of persistently active infection without an obvious explanation [16] and death due to any cause during hospitalization was referred to as in-hospital mortality. The severity of bacteremia when first presented at our hospital was graded by the Pittsburgh bacteremia score, which was based on the evaluation of mental status, body temperature, blood pressure, need for mechanical ventilation and presence or absence of cardiac arrest, and critical illness was defined as a score of at least 4 points [17]. Empirical antimicrobial therapy was considered to be appropriate, if the etiological pathogen was susceptible in vitro to at least one of the drugs administered within 3 days after the onset of bacteremia [18].

### Liquid-toxic (LT) assay of *C. elegans* infected by aeromonads

The virulence of 36 blood isolates of four *Aeromonas* species were tested by the LT assay of *C. elegans*. The detailed procedures for LT assays were described elsewhere [12]. In brief, the survival rate of worms in LT assay was determined by counting the number of live worms out of the total number of worms under a dissecting scope. The mean survival rates of *C. elegans* from day 1 to day 3 were determined for four *Aeromonas* species. LT assay procedures are detailed in the File S1.

### Cytotoxicity assay

Cytotoxicity assays were performed in a mouse C2C12 fibroblast cell line (American Type Culture Collection No.: CRL-1772; BCRC no.: 60083) obtained from the Bioresource Collection and Research Center, Hsinchu, Taiwan. The cells were cultured in a complete medium consisting of Dulbecco's Modified Eagle's medium (DMEM, Gibco, Grand Island, NY, USA) and 10% fetal bovine serum (FBS, Invitrogen, Carlsbad, CA, USA). All cells were incubated in 10-cm tissue culture dishes (BD Falcon, San Jose, CA, USA) at 37°C and 5% CO2. They were ready for use after cultivation for at least 2–3 days. The *Aeromonas* isolates were grown in 1 ml of LB medium for 3 hours, and 0.5 ml of the bacterial solution was transferred to 15 ml disposable tubes and cultivated for another 17 hours at 37°C. The C2C12 fibroblast cells were separated by centrifugation and seeded into 96-well plates (1×10^4^ cells/well). The cells were incubated with bacterial cultures at a multiplicity of infection (MOI) of 25. After incubation at 37°C for 2 hours, the culture medium was examined for the release of lactate dehydrogenase (LDH) by a CytoTox 96 kit (Promega, Madison, WI). A 0.1% Triton X-100 solution was used as a positive control, and serum-free Roswell Park Memorial Institute (RPMI) medium (GIBCO^®^, Grand Island, N.Y., USA) was used as a negative control. The cytotoxicity activity was expressed as the mean of triplicate measurements of released LDH levels compared with Triton X-100 exposure (defined as 100% cytotoxicity).

### Life span and pathology of BALB/c mice with *Aeromonas* intramuscular infection

Three clinical blood isolates of each species, i.e. *A. dhakensis*: A2-061, A2-094, A2-107; *A. hydrophila*: A2-011, A2-066, A2-078; *A. caviae*: A2-9307121, A2-961204, A2-9310251; *A. veronii*: A2-007, A2-029, A2-041, were randomly selected for the mouse study. All the isolates of the same species were genetically different as evidenced by the gel profiles of arbitrarily primed polymerase chain reaction (AP-PCR) methods [19] (data not shown). Six to ten week-old female BALB/c mice weighing 18–22 grams were obtained from National Laboratory Animal Center. Animals were housed in a pathogen-free environment using 12 h alternating periods of light and dark until the initiation of experiments. Each mouse was injected intramuscularly at the right thigh with 100 ìL containing 2.5×10^6^ colony forming unit (CFU) of *Aeromonas* isolates. Seven mice were tested for each isolate. At 24 h after injection, one mouse was sacrificed for pathological examination. The infected soft tissues were dissected and fixed in 10% v/v neutral-buffered formalin, and then stained with hematoxylin and eosin for light microscopy.

The severity of soft tissue damage was evaluated according to the extent of inflammatory cell infiltration, edema, or myonecrosis based on a semi-quantitative score designed for evaluating peripheral compartment syndrome [20]. In brief, five high-powered (100x) representative fields were scored by a blinded pathologist based on the following criteria, including items of inflammatory cell infiltrate (1, inflammatory cell penetration into <10% of muscle parenchyma; 2, 11%–50% of parenchyma; 3,>50% of parenchyma; edema (1, edema visible in <10% of muscle parenchyma; 2, 11%–50% of parenchyma; 3,>50% of muscle parenchyma; and myonecrosis (1, abnormal muscle fibers in <10%; 2, 11%–50%; 3,>50% of views), with a sum score range of 3 to 9. The survival of six mice was monitored daily for 14 days.

All the animal experiments in this study were carried out in strict accordance with the recommendations in the Guidelines for Committee of Laboratory Care and Use, developed by the National Cheng Kung University. The protocol was ethically approved by the Institutional Animal Care and Use Committees and the National Cheng Kung University (Permit No, 101050). Experiments were planned and conducted with environmental enrichment, veterinary oversight and the use of appropriate analgesics and anesthesia when needed. All animals were monitored daily by trained personnel. The frequency of monitoring was increased when animals developed or if they were anticipated to develop clinical signs of severe sepsis. In the study, the animals were humanely sacrificed when they met either the criteria of hypotheremia <34 °Cor >20% body weight loss. Monitoring body temperature was accomplished using laser directed infrared temperature scanners. Intramuscular inoculation with pathogens was performed under anesthesia by inhalation of 2% isoflurane with 1.5L/min oxygen, and all efforts were made to minimize suffering. Euthanasia at the completion of experiments was carried out by exsanguination, under deep anesthesia with inhalation of 3% isoflurane with 1.5L/min oxygen.

### Exoenzyme assay

Qualitative assays of exoprotease activity were performed on LB agar containing 2% (wt/vol) skimmed milk (Difico Laboratories, Detroit, MI, USA). Hemolytic activity was assayed on LB agar containing 5% (vol/vol) sheep blood, amylase activity on starch agar (Difco Laboratories, Detroit, MI, USA), and nuclease activity on DNase agar with methyl green (Difco Laboratories, Detroit, MI, USA). A single streak of undiluted organisms were inoculated on blood agar plates and incubated at 37°C for 24 hours, and on starch and DNase agar plates for 48 hours. Positive reactions for exoprotease and hemolytic tests were the presence of clear zones surrounding the streaks. Amylase activity was examined by removing growth from each streak to expose the agar plates to Gram iodine. Starch hydrolysis was indicated by a colorless zone surrounding colonies. *Bacillus subtilis* ATCC^T^ 6633 and *Escherichia coli* ATCC^T^ 25922 were positive and negative control strains for the amylase test, respectively. For the DNase test agar with methyl green, positive reactions were identified as decolorization around the streaks. Positive and negative control strains for the DNase test were *Staphylococcus aureus* ATCC^T^ 25923 and *Staphylococcus epidermidis* ATCC^T^ 12228, respectively.

### PCR detection of the genes encoding putative virulence factors

Polymerase chain reactions (PCRs) using previously described primers and conditions were conducted to detect the genes encoding heat-stable enterotoxin (*ast*), hemolysin (*ahh1*), aerolysin (*aerA*), components of the type III secretion system (TTSS) (*ascV*), or ADP-ribosylating toxin (*aexT*) [21]. *A. hydrophila* ATCC 7966^T^ was used as a positive control for *ahh1*, *aerA* and *ast* [21], [22] and *A. veronii* ATCC 9071^T^ a positive control for *ascV* and *aexT* [12].

### Statistical analysis

Statistical analyses were performed to compare the variables among the adults infected by different *Aeromonas* isolates. Categorical variables were compared by the Chi-square test or Fisher's exact test, if the expected counts were less than 5. Cytotoxicity was compared by one-way analysis of variance (ANOVA) with Turkey's HSD (Honestly Significantly Difference) post hoc test. The scores for muscle damage in BALB/c mice were compared by the Kruskal-Wallis one-way analysis of variance (ANOVA) with Dunn's post hoc test. Mouse survivals were analyzed by the log-rank test. Data were analyzed by the software of GraphPad Prism, version 5.01 (PraphPad Software Inc. California, USA).

## Results

Clinical features of patients with *Aeromonas* bacteremia were summarized in Table 1. Polymicrobial infection was more common in patients with *A. veronii* bacteremia (*P* = 0.032). Patients with *A. dhakensis* bacteremia tended to have liver cirrhosis (*P* = 0.029). The sources of *Aeromonas* bacteremia were identified in 36.1% (13) of 36 patients, including vascular catheter-related infections (4), spontaneous bacterial peritonitis (3), necrotizing fasciitis (2), biliary tract infections (2), pleural empyema (1), and appendicitis (1). Four (44.4%) of 9 patients with *A. dhakensis* bacteremia empirically received *in vitro* active antimicrobial agents, in contrast to 20 (74.1%) of 27 patients with non-*dhakensis Aeromonas* bacteremia (*P* = 0.12, Fisher's exact test). The proportion of critical illness, *i.e.* Pittsburgh bacteremia score ≥4, was similar among the patients with bacteremia due to four *Aeromonas* species.

**Table 1:** Clinical features of patients with septicemia caused by different *Aeromonas* species.

Characteristics	No. (%) of patients				*P* values
	*A. dhakensis* n = 9	*A. hydrophila* n = 9	*A. veronii* n = 9	*A. caviae* n = 9	
Age ≥60 year-old	3 (33.3)	7 (77.8)	5 (55.6)	5 (55.6)	0.308
Male gender	8 (88.9)	6 (66.7)	5 (55.6)	4 (44.4)	0.239
Monomicrobial bacteremia	8 (88.9)	8 (88.9)	3 (33.3)	6 (66.7)	0.032
Source of infection					
Primary bacteremia	6 (66.7)	6 (66.7)	6 (66.7)	5 (55.6)	0.948
Secondary bacteremia	3 (33.3)	3 (33.3)	3 (33.3)	4 (44.4)	
Spontaneous bacterial peritonitis	2	1	-	-	
Vascular-catheter related infection	-	1	-	3	
Necrotizing fasciitis	1	1	-	-	
Others	-	-	3&	1$	
Underlying diseases					
Liver cirrhosis	6 (66.7)	3 (33.3)	3 (33.3)	0	0.029
Active malignant diseases	1 (11.1)	4 (44.4)	2 (22.2)	4 (44.4)	0.316
Pittsburgh bacteremia score ≥4	2 (22.2)	2 (22.2)	0	1 (11.1)	0.465
Appropriate empirical antibiotics	4 (44.4)	6 (66.7)	9 (100)	5 (55.6)	0.06
Mortality					
Sepsis-related	3 (33.3)	0/8*	0	0	0.024
In-hospital	5 (55.6)	0/8*	1 (11.1)	0	0.004

*One patient with necrotizing fasciitis was transferred to another hospital.

& Biliary tract infection, pleural empyema, appendicitis.

$ Biliary tract infection.

The sepsis-related and in-hospital mortality rates of patients with *A. dhakensis* bacteremia were significantly higher than those of bacteremia caused by non-*dhakensis Aeromonas* species (*P* = 0.024 and 0.004, respectively). Even taking monomicrobial *Aeromonas* bacteremia (i.e. 8 episodes of *A. dhakensis* bacteremia, 7 *A. hydrophila*, 3 *A. veronii*, and 6 *A. caviae*) into consideration, the sepsis-related (37.5% *vs.* 0%, *P* = 0.028) or in-hospital mortality rate (50% *vs.* 0%, *P* = 0.007) of *A. dhakensis* bacteremia remained higher than that of monomicrobial bacteremia due to non-*dhakensis Aeromonas* species. Furthermore, the patients with *A. dhakensis* bacteremia and appropriate empirical therapy fared worse than those with non-*dhakensis Aeromonas* bacteremia and appropriate empirical therapy (14-day sepsis-related mortality rate: 2/4, 50% *vs.* 0/20, 0%; *P* = 0.02). Among three fatal patients with *A. dhakensis* bacteremia, each had severe underlying disease (*i.e.* liver cirrhosis in two patients and end-stage renal disease 1). Two had received appropriate empirical antibiotic therapy, but expired within 7 days after the onset of bacteremia.

**Figure 1:**
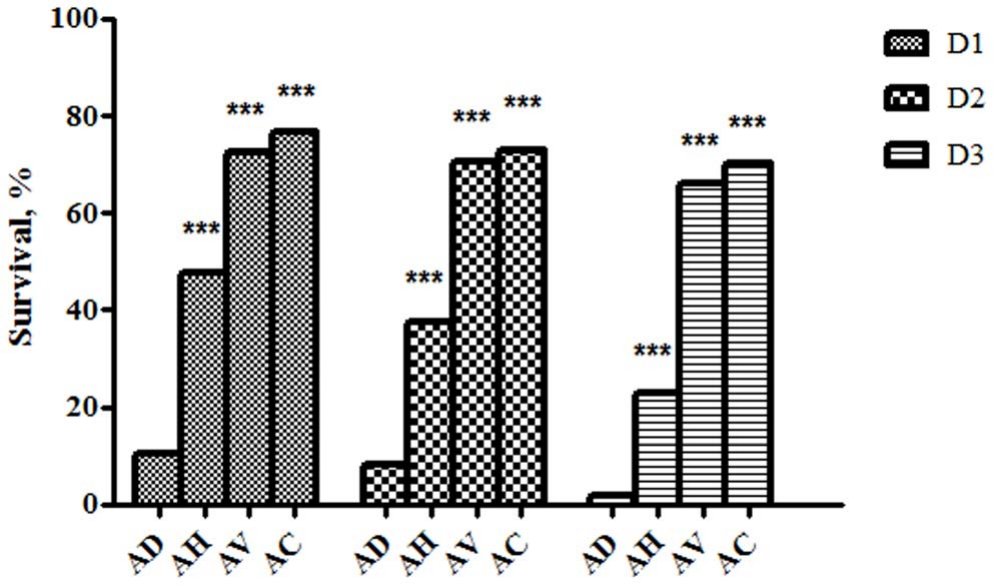
Three-day survivals of *Caenorhabditis elegans* co-cultivated with *Aeromonas* isolates of different species: *Aeromonas dhakensis* (AD), *Aeromonas hydrophila* (AH), *Aeromonas veronii* (AV), and *Aeromonas caviae* (AC) in the liquid-toxic assay. ***P<0.0001, as compared with AD.

In the LT assays, 3-day survivals of *C. elegans* co-cultivated with *Aeromonas* isolates and *E. coli* strain OP50, which was a food source for *C. elegans* and used as the control, were shown in Figure 1. The survival rates of *C. elegans* fed with *A. dhakensis* within the first three days were significantly lower than those with *A. hydrophila*, *A. veronii*, and *A. caviae* (all *P* values <0.0001). The worms fed with *A. caviae* showed a higher survival rate than that fed with the other *Aeromonas* species (all *P* values <0.0001).

**Figure 2:**
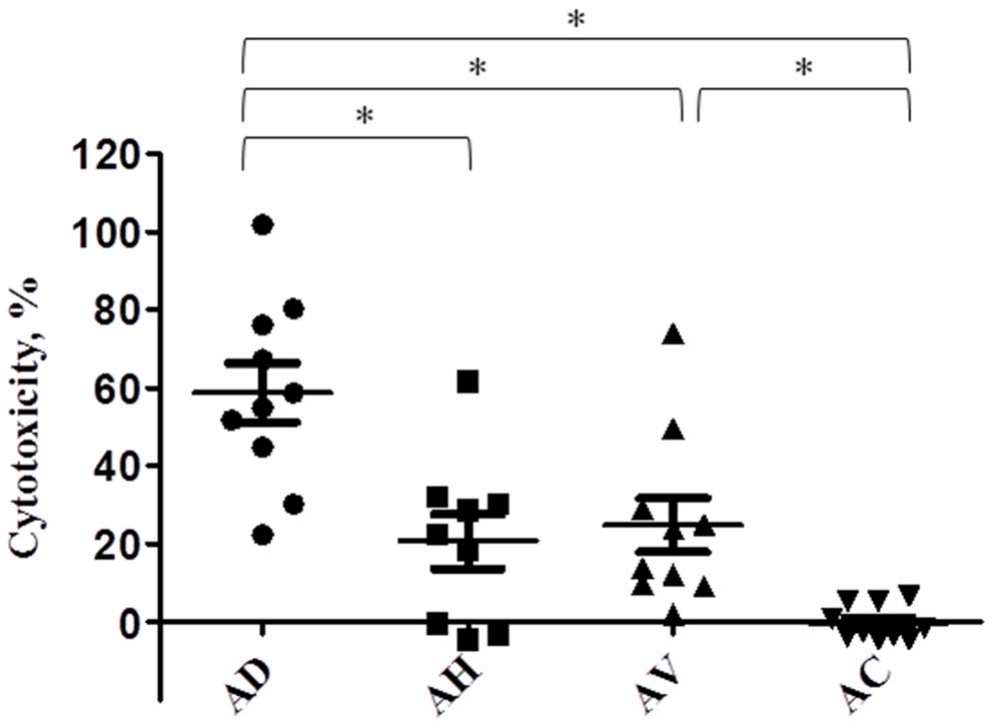
Cytotoxicity of *Aeromonas dhakensis* (AD, n = 9), *A. hydrophila* (AH, n = 9), *A. veronii* (AV, n = 9) and *A. caviae* (AC, n = 9) isolates to C2C12 mouse fibroblast cell lines, which is expressed as the proportions of the released LDH levels induced by *Aeromonas* isolates, as compared with the LDH level by 0.1% Triton X-100 (a positive control: 100%). *P<0.05.

Cytotoxicity of a total of 36 *Aeromonas* isolates of four species was assessed in C2C12 mouse fibroblast cell line. The mean values ± standard errors of the released LDH levels induced by *Aeromonas* isolates as compared with the LDH level by 0.1% Triton X-100 (a positive control: 100%), were 58.8±8.4% by *A. dhakensis*; 20.5±7.1% by *A. hydrophila*; 26.1±7.6% by *A. veronii*, and, −0.39±1.5% by *A. caviae* (Figure 2) (one-way ANOVA test, *P* = 0.0001). Post-Hoc Turkey's HSD test demonstrated *A. dhakensis* isolates exhibited more potent cytotoxicity than other species (all *P* values <0.05), and *A. veronii* isolates higher cytotoxicity than *A. caviae* (*P*<0.05) to the C2C12 cell line.

**Figure 3:**
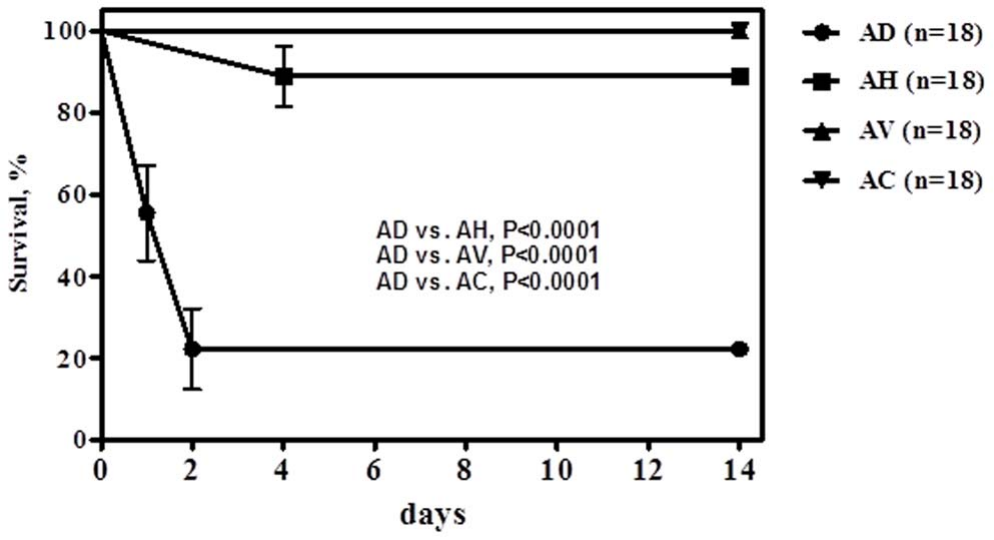
Life spans of BALB/c mice infected by *Aeromonas* isolates of four *Aeromonas* species. Three isolates of each species were tested, and six mice were infected by each isolate. A volume of 100 ìL Luria-Bertani broth containing 2.5×10^6^ colony forming units was injected intramuscularly at right thigh of BALB/c mice.

The life spans of the mice infected by different *Aeromonas* species intramuscularly was shown in Figure 3. After two weeks, of 18 mice infected by *A. dhakensis*, only four (22.2%) survived. Of note 14 mice expired within 48 hours. In contrast, 16 (88.9%) of 18 mice infected by *A. hydrophila* and all by *A. veronii* or *A. caviae* survived for 14 days (all *P* values <0.0001). Kruskal–Wallis one-way ANOVA with Dunn's post hoc test revealed that a similar severity of muscle damage at the inoculated sites after 24 hours of infection was discerned in the mice infected by *A. dhakensis*, *A. hydrophila*, and *A. veronii* (see Figure S1). The severity of muscle damage induced by *A. caviae* infection was significantly milder than that by *A. hydrophila* (*P*<0.05). Pathological characteristics of muscle tissue damage, such as fragmentation of muscle fibers, edema of myocytes, and infiltration of inflammatory cells, were rarely seen in mice with *A. caviae* infection (see Figure S2).

The results of agar plate assays for phenotypic activity of exoenzymes, including exoprotease, amylase, and DNase, were demonstrated in Table 2. The proportion of hemolytic phenotype in the *A. caviae* isolates was 44.4%, which was significantly lower than the other species (*P* = 0.001). Of four *Aeromonas* species, the activity of amylase, DNase, and exoprotease was present in most of the isolates.

**Table 2:** The results of agar plate assays for exoprotease, amylase, DNase, and hemolytic activity of isolates of four *Aeromonas* species: *A. dhakensis*, *A. hydrophila*, *A. veronii* and *A. caviae*.

	Isolate number (%)				*P* value
	*A. dhakensis*, n = 9	*A. hydrophila*, n = 9	*A. veronii*, n = 9	*A. caviae*, n = 9	
Exoprotease	9 (100)	9 (100)	9 (100)	7 (77.8)	0.096
Amylase	9 (100)	9 (100)	8 (88.9)	8 (88.9)	0.548
DNase	9 (100)	9 (100)	8 (88.9)	9 (100)	0.379
Hemolysis*	9 (100)	9 (100)	9 (100)	4 (44.4)	0.001

*All positive isolates showed β-hemolysis.

**Figure 4:**
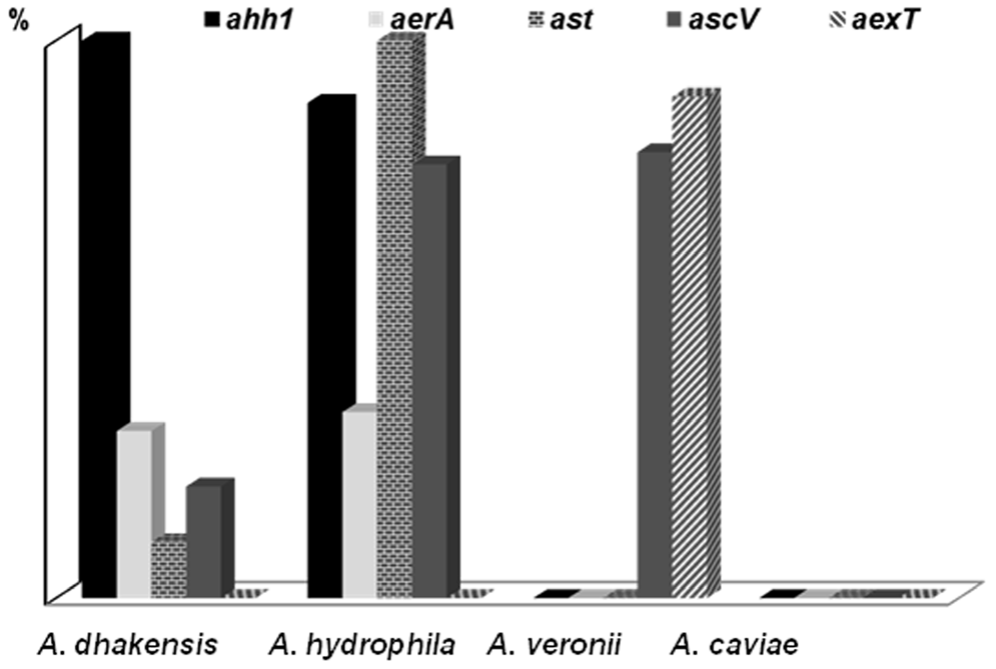
Distribution of putative virulence factors among blood isolates of four *Aeromonas* species: *Aeromonas dhakensis* (n = 9), *Aeromonas hydrophila* (n = 9), *Aeromonas veronii* (n = 9), and *Aeromonas caviae* (n = 9).

The genetic distribution of virulence factors among *Aeromonas* blood isolates was summarized in Figure 4. In all *A. dhakensis* and *A. hydrophila* isolates, *ahh1* was detected, and *aerA* in 33.3% of both *A. dhakensis* and *A. hydrophila* isolates, respectively. However, *ahh1* and *aerA* were not found in *A. veronii* and *A. caviae*. Of note, *aexT* was only identified in *A. veronii* isolates and none of *A. caviae* isolates possessed any of five tested genes. Among bacteremic isolates of *A. hydrophila* and *A. dhakensis*, *ast* (100% *vs.*11.1%, *P<*0.0001) was primarily present in *A. hydrophila* isolates.

## Discussions

In the present study, the mortality rate of the patients with *A. dhakensis* bacteremia was higher than that of bacteremia due to non-*dhakensis Aeromonas* species (33.3%, 3/9 *vs.* 0%, 0/26; *P* = 0.001), and the difference remained significant, if only those with appropriate empirical therapy were taken into consideration (2/4, 50% *vs.* 0/20, 0%; *P* = 0.02). Therefore, our results reminded the clinicians that *A. dhakensis* infections can be life-threatening in susceptible hosts, despite of early appropriate antimicrobial therapy. In addition, our work provided more *ex vivo* and *in vivo* evidences of the potent virulence of *A. dhakensis*, and such a finding was in accordance with the clinical findings. *A. dhakensis* has been reported to be more toxic than other species to human blood cell lines [11], and wound isolates of *A. dhakensis* had been demonstrated to be more virulent in the *C. elegans* model and human normal skin fibroblast cells than wound isolates of *A. hydrophila* [12]. However, the prevalence of human infections caused by *A. dhakensis* is often underestimated due to the misidentification as *A. hydrophila* by current phenotype-based identification schemes. Several reports indicated that the isolates phenotypically identified as *A. hydrophila* were *A. dhakensis*, if *rpoD* or *gyrB* was sequenced [10], [11]. Thus, with the potent virulence of *A. dhakensis*, it is justified to precisely differentiate *A. dhakensis* from other *Aeromonas* species.

In the sepsis-related mortality rate of 9 cases of *A. dhakensis* bacteremia, 33.3%, were comparable with that of *A. hydrophila* bacteremia in the literature (33–36%) [5], [8], [9]. However, none of the 9 cases of bacteremia in our study died of sepsis due to *A. hydrophila*. As mentioned before, phenotypically identified *A. hydrophila* was actually *A. dhakensis* by molecular methods. Therefore, it is not surprising that the clinical outcome of published cases of *A. hydrophila* is similar to that of *A. dhakensis*. In addition to the significant virulence of *A. dhakensis*, other clinically relevant information from the present work is the low virulence of *A. caviae*. The heterogeneous distribution of virulence genes in bacteremic *Aeromonas* isolates of four species may partially account for the virulence comparative results in animals or patients. The so-called “virulent species” in the present study, i.e. *A. dhakensis*, *A. hydrophila*, or *A. veronii*, harbored an array of virulence factors, such as hemolysin (*ahh1*), aerolysin (*aerA*), cytotoxin (*ast*), type III secretion system (*ascV* and *ascF-G*) [11], [23], [24].

*A. veronii* and *A. caviae* bacteremia have been associated with a high mortality, for example 42% in *A. veronii* bacteremia in Taiwan [5], and 20% and 17% in *A. veronii* and *A. caviae* bacteremia, respectively, in Japan [25]. The difference of mortality rates between studies may be related to the heterogeneous study population. Of our 36 cases, only 33.3% had liver cirrhosis. In contrast, of 154 cases in another Taiwanese study, 64.3% had liver cirrhosis [5], and in a Japanese study 36.1% of 36 cases had chronic hepatic disease [25]. Moreover, in the two published studies, the accuracy of species identification based on traditional biochemical tests was questionable. Therefore, generalization of our conclusions to other hospitals or areas should be cautious.

A correlation between the virulence and hemolytic activity of aeromonads has been proposed [22]. The production of hemolysin or aerolysin in aeromonads has been related to their pathogenic potential in hosts [26]–[29] and inactivation of aerolysin and hemolysin genes in *A. hydrophila* attenuates the pathogenicity in wound and systemic infection models of mice [26], [27]. Hemolysin (*ahh1*) or aerolysin genes (*aerA*) were not found in *A. caviae* isolates, as reported by Osman *et al.* in their *Aeromonas* isolates from retail meats in Egypt [30]. In addition, the genes encoding other important virulence factors, such as cytotoxin (*ast* and *alt*) [31] or TTSS genes (*ascV* and *aexT*) [21], were rarely found in *A. caviae* isolates. These results are in accordance with the impression that *A. caviae* is less invasive in humans and animal models. Comparisons of genetic information from whole genome sequences of clinical *Aeromonas* strains may identify potential genetic traits responsible for virulence [32].

*Aeromonas* skin and soft-tissue infections often were polymicrobial infections after exposure to aquatic environments [12], in immunocompromised subjects with liver cirrhosis [33], chronic renal failure, or malignancy [6], [7], [34]. The precise contribution of *Aeromonas* species or host factors to the severity of skin and soft-tissue infection is difficult to estimate in the real world. Animal models with controlled environmental and host variables may be used to compare the pathogenicity between species. Several animal models have been proposed for studying *Aeromonas* infections. These models, including leech, blue gourami, zebrafish, amoebae, nematode, or mice, had distinct advantages to link the pathogenicity in human [2]. We had demonstrated that the *C. elegans* LT assay is a plausible model to study the virulence of aeromonads, with several experimental advantages, such as a short round time, rapid generation time, large progeny, and ease of observation [12], [35].

In the mice with intramuscular infection, though *A. dhakensis*, *A. hydrophila* and *A. veronii* isolates can all cause extensive tissue damage at the initial 24 hours, *A. dhakensis* infections lead to more fatality in mice at 2 weeks. These results suggest efficient adaption of *A. dhakensis* to the host immune or more pathogenicity to mice. Such a mouse model with intramuscular infection has been used by Grim et al. to evaluate the pathogenicity of different genotypes of *A. hydrophila* [36]. Therefore, it is possible that the mouse model of intramuscular infection could be a research platform to investigate the virulence signatures of *Aeromonas* species in human infections.

Moreover, the toxicity difference among varied *Aeromonas* species, i.e. invasive species like *A. dhakensis* and less invasive species as *A. caviae* in *C. elegans* LT assay, was in accordance to those findings in the BALB/c mouse model, which is a feasible mammalian model to investigate the pathogenicity of *Aeromonas* species in soft-tissue infections. The majority (78%) of mice intramuscularly infected by *A. dhakensis* died within 48 hours and the degree of inflammatory response in mouse muscles was less severe in *A. caviae* than other species on pathological examination. The survival outcomes in BALB/c mice with intramuscular infections are compatible to the poor prognosis in necrotizing fasciitis, myonecrosis, or severe soft-tissue infections due to *A. dhakensis*, *A. hydrophila*, or *A. veronii* in clinical reports [6], [12], [34], [37]–[39]. These findings suggest virulence variation among *Aeromonas* species.

There are several limitations in the present study. Firstly, all the isolates were collected from a medical center, and therefore the caveat is that interpretations from our results may not be generalized to other areas. For example, the prevalence of virulence genes among *A. caviae* isolates here is low. In contrast, clinical *A. caviae* stool isolates from Spain and Mexico may carry aerolysin and hemolysin genes, with a prevalence of 96.0% and 84.2%, respectively [24]. Nevertheless, all reports together suggested the geographical genetic variation not only in environmental but also in clinical aeromonads. Secondly, our case number of *Aeromonas* bacteremia is too limited to represent the clinical outcome of *Aeromonas* bacteremia due to different species. A clinical study including more cases of *Aeromonas* bacteremia is ongoing to disclose the virulence variation of *Aeromonas* species. Nevertheless, our study highlights that correct identification of *A. dhakensis* among *Aeromonas* isolates is of clinical value due to its potential virulence. Third, the possibility of underestimation of infection sources should be considered, because clinical data in this study was obtained from the retrospective review of medical charts. However, the identification rate of foci of *Aeromonas* bacteremia was 36.1%, which was comparable to those reported in two published reports in Taiwan (43.3% and 48.5%) [9], [40].

In conclusion, clinical data, *ex vivo* experiments, and animal studies suggest there is virulence variation among clinically important *Aeromonas* species. More clinical investigations and laboratory work are warranted to compare the pathogenicity of *Aeromonas* species in human infections

## Supporting Information

Figure S1 Pathological scores of soft-tissue damage at 24 hours following inoculation with 100 µL of Luria-Bertani solution containing 2.5×10^6^ colony forming units of four *Aeromonas* species, *i.e.*, *A. caviae*, *A. hydrophila*, *A. veronii*, and *A. dhakensis*, over right thigh of BALB/c mice. There are three isolates of each species for the test. The infected soft tissues of mice were dissected and fixed in 10%v/v neutral-buffered formalin, and then stained with haematoxylin and eosin for light microscopy.
(TIF)

Figure S2 Fragmentation (arrows) of myocytes, inflammatory cells infiltration (arrowheads), and edema (dashed arrows) of muscle parenchyma were observed in high-powered fields (100x) of hematoxylin and eosin staining of infected muscle of BALB/c mice with inoculation of 4 *Aeromonas* species for 24 hours (A, *A. caviae*; B, *A. hydrophila*; C, *A. veronii*; D, *A. dhakensis*).
(TIF)

File S1 (DOCX)
