# Supplementary figures and images for: Virulence Diversity among Bacteremic Aeromonas Isolates: Ex Vivo, Animal, and Clinical Evidences
Source: PLoS One. 2014 Nov 6;9(11):e111213. doi: 10.1371/journal.pone.0111213 (PMC4222899; doi:10.1371/journal.pone.0111213)

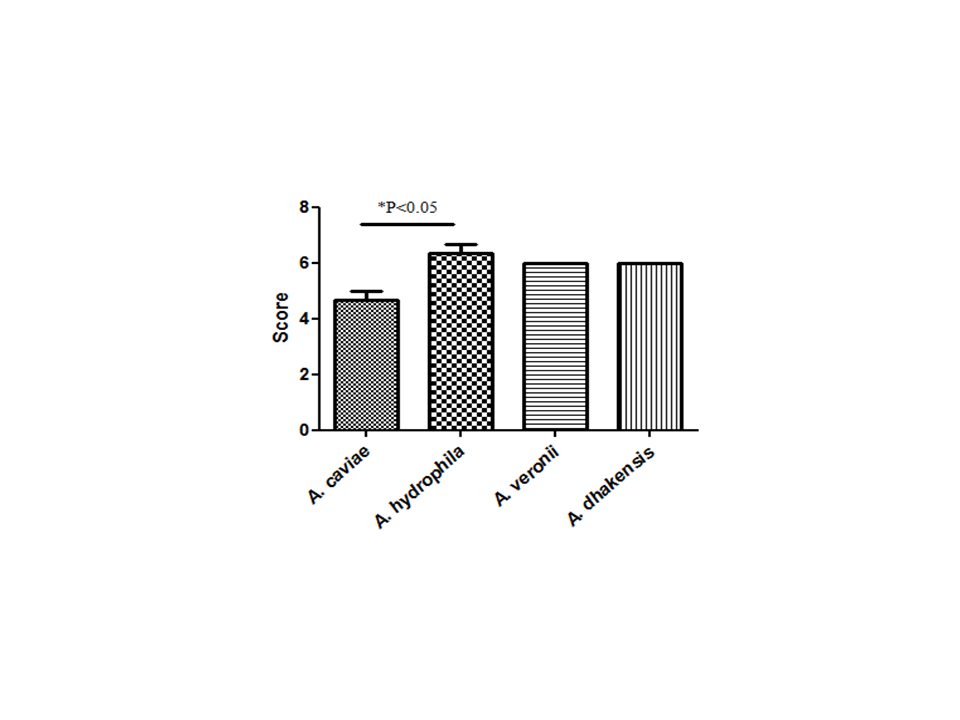

Supplement: Figure S1 — Pathological scores of soft-tissue damage at 24 hours following inoculation with 100 µL of Luria-Bertani solution containing 2.5×10^6^ colony forming units of four Aeromonas species, i.e., A. caviae, A. hydrophila, A. veronii, and A. dhakensis, over right thigh of BALB/c mice. There are three isolates of each species for the test. The infected soft tissues of mice were dissected and fixed in 10%v/v neutral-buffered formalin, and then stained with haematoxylin and eosin for light microscopy. (TIF) [file pone.0111213.s001.tif]

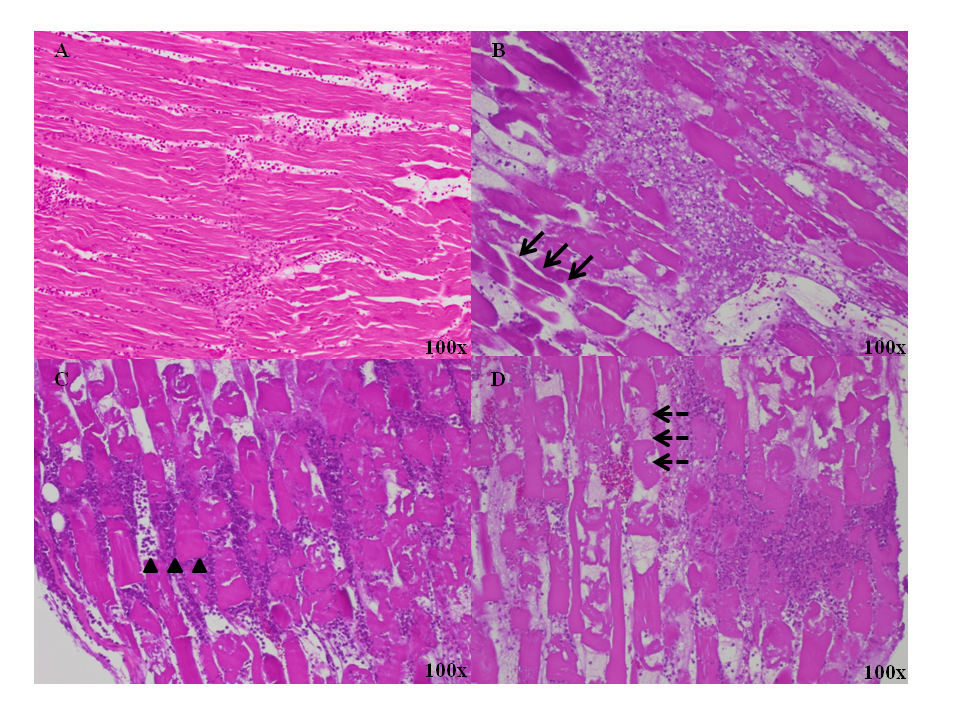

Supplement: Figure S2 — Fragmentation (arrows) of myocytes, inflammatory cells infiltration (arrowheads), and edema (dashed arrows) of muscle parenchyma were observed in high-powered fields (100x) of hematoxylin and eosin staining of infected muscle of BALB/c mice with inoculation of 4 Aeromonas species for 24 hours (A, A. caviae; B, A. hydrophila; C, A. veronii; D, A. dhakensis). (TIF) [file pone.0111213.s002.tif]
