# Supplementary material for: Virulence Diversity among Bacteremic Aeromonas Isolates: Ex Vivo, Animal, and Clinical Evidences
Source: PLoS One. 2014 Nov 6;9(11):e111213. doi: 10.1371/journal.pone.0111213 (PMC4222899; doi:10.1371/journal.pone.0111213)
Supplement: File S1 — (DOCX) [file pone.0111213.s003.docx]

**Table S1.** The GenBank/DDBJ accession no. of *rpoD* sequences for 36 *Aeromonas* isolates: 9 *A. dhakensis*, 9 *A. hydrophila*, 9 *A. veronii,* and 9 *A. caviae*.

| Species | Accession no. | Isolate no. |
| --- | --- | --- |
| *A. dhakensis* | AB904676 | A2-005 |
|  | AB904677 | A2-033 |
|  | AB904678 | A2-070 |
|  | AB904679 | A2-042 |
|  | AB904681 | A2-061 |
|  | AB904682 | A2-094 |
|  | AB904683 | A2-107 |
|  | AB904684 | A2-157 |
|  | AB904685 | A2-155 |
| *A. hydrophila* | AB904686 | A2-011 |
|  | AB904687 | A2-066 |
|  | AB904688 | A2-078 |
|  | AB904689 | A2-9404091 |
|  | AB904690 | A2-9411261 |
|  | AB904691 | A2-960701 |
|  | AB904692 | A2-048 |
|  | AB904693 | A2-970201 |
|  | AB904694 | A2-970410 |
| *A. veronii* | AB904695 | A2-007 |
|  | AB904696 | A2-029 |
|  | AB904697 | A2-041 |
|  | AB904698 | A2-9406151 |
|  | AB904699 | A2-9508181 |
|  | AB904700 | A2-9512301 |
|  | AB904701 | A2-960402 |
|  | AB904702 | A2-960904 |
|  | AB904703 | A2-970204 |
| *A. caviae* | AB904705 | A2-074 |
|  | AB904706 | A2-108 |
|  | AB904707 | A2-9410281 |
|  | AB904708 | A2-970703 |
|  | AB904709 | A2-971106 |
|  | AB904710 | A2-9307121 |
|  | AB904711 | A2-961204 |
|  | AB904712 | A2-9310251 |
|  | AB904714 | A2-9312092 |

**Materials and Methods**

**Liquid toxicity (LT) assay of *Caenorhabditis elegans***

The bacteria grown in LB for 18-24 h at 37℃ and standardized to an OD_600_ of 3.0 were prepared for the assay. The *C. elegans* strains, the wild-type Bristol strain N2 were provided by the *Caenorhabditis* Genetics Center, which is funded by the NIH National Center for Research Resources. The *C. elegans* strains were maintained on nematode growth medium (NGM) agar plates (prepared as described at http://www.wormbook.org/, accessed 01 August 2014) using *Escherichia coli* strain OP50 as the food source. The synchronized adult L4 worms on NGM plates were washed in M9 buffer (prepared as described at http://www.wormbook.org/, accessed 01 August 2014). After centrifugation, the pellets of worms were re-suspended in 5 μl of S medium (prepared as described at http://www.wormbook.org/, accessed 01 August 2014), and approximately 30-40 worms were placed in a 48-well plate with 5 μl of fluorodeoxyuridine (Sigma-Aldrich^®^, Saint Louis, Missouri, USA) to prevent reproduction. Finally, 190 μl of bacteria in LB solution was added to give a final volume of 200 μl for each lawn. The assay plates were incubated at 25℃ and observed for 3 days.
